# East Texas Medico-Chirurgical Society

**Published:** 1901-01

**Authors:** E. W. Guinn

**Affiliations:** Secretary, Rusk, Texas


### East Texas Medico-Chirurgical Society.

RUSK, TEXAS, December 21, 1900.

*Editor Texas Medical Journal:*

The East Texas Medico-Chirurgical Society met in regular session in the city of Palestine, November 28 and 29, 1900, with a fair attendance. The President being absent, Vice-President J. H. Evans presided. There was great interest and enthusiasm manifested in the discussion of papers. And I am sorry I can not report the discussions in detail, the papers I send you for publication. There were several new members admitted. Dr. J. J. Sprill, of Jewett, was appointed Chairman on the section of General Medicine; Dr. J. S. Waters, of Crockett, Chairman of Section on General Surgery; Dr. C. S. Lane, of Jacksonville, Chairman of Section of Obstetrics and Gynecology and Dr. W. J. W. Smith, of Nacogdoches, Chairman of Section of Diseases of Children and Therapeutics. Drs. O. L. Hethcock, E. W. Link and E. B. Parsons, Palestine, Committee of Arrangements for next meeting, May, 1901.

It is earnestly hoped that every physician in East Texas will attend.

E. W. Guinn, M. D.,
Secretary, Rusk, Texas.

